# Supplementary material for: Comparative multiomics analysis of cell physiological state after culture in a basket bioreactor
Source: Sci Rep. 2022 Nov 23;12:20161. doi: 10.1038/s41598-022-24687-4 (PMC9686226; doi:10.1038/s41598-022-24687-4)

23666041.1:51267146:51267197:-@NW_023666041.1:51267291:51268043:-@NW_023666041.1:51267901:5126[illegible]

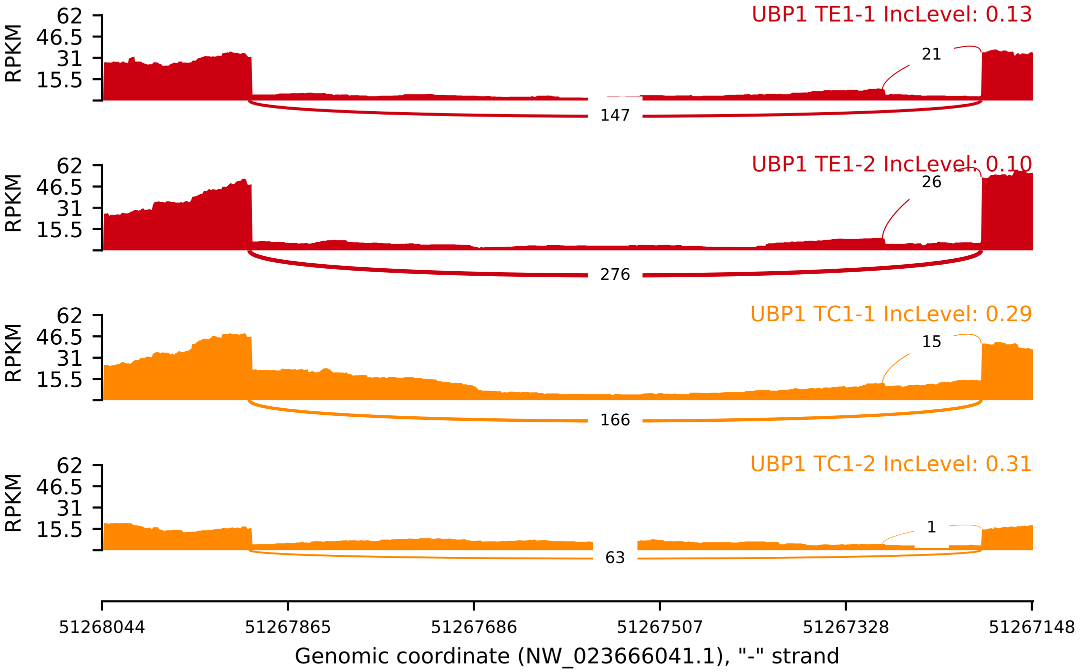

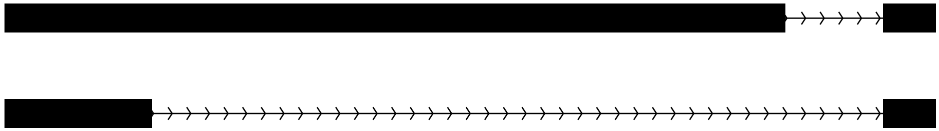

Supplement: Supplementary file 1 — Supplementary Information 1. [file 41598_2022_24687_MOESM1_ESM.zip › raw data/Transcriptomic raw data/7.AS/2.dasplot/TE1vsTC1/A5SS/12_UBP1_NW_023666041.1_51267146_51267197_-@NW_023666041.1_51267291_51268043_-@NW_023666041.1_51267901_51268043_-.pdf]
